# Editorial: Undergraduate Education for Public Health in the United States

**DOI:** 10.3389/fpubh.2015.00138

**Published:** 2015-05-26

**Authors:** Cheryl L. Addy, Daniel S. Gerber, David T. Dyjack, Connie J. Evashwick

**Affiliations:** ^1^University of South Carolina, Columbia, SC, USA; ^2^University of Massachusetts at Amherst, Amherst, MA, USA; ^3^National Environmental Health Association, Denver, CO, USA; ^4^George Mason University, San Diego, CA, USA

**Keywords:** editorial, public health education, public health education in USA, undergraduate public health education, public health curriculum

Undergraduate education focused on public health (UGPH) has burgeoned over the past decade (1, 2). While this trend is widely acknowledged, a critical analysis of drivers, constructs, and implications is missing from the published literature. The Research Topic comprising articles on UGPH attempts to address this gap in knowledge by providing descriptions of exemplary programs, curriculum recommendations, and commentaries on career relevancy for the future workforce.

Our aim is to advance the field as more students, faculty, and universities explore how best to launch and integrate public health into the education of undergraduate college students. Moreover, we hope that those faculties and universities that have been engaged in public health undergraduate education for many years will recognize the contribution that they can make by documenting, disseminating, and re-examining their work. To the extent that we have become an evidence-driven society, the need for data is compelling. The academic enterprise would benefit from descriptive data that describe the landscape of the field as a basis for subsequent evaluation studies, research on pedagogy, and concrete information on career trajectories.

We note three caveats about this compilation. First, we limited this collection of articles to those focused tightly on undergraduate education for the general public health degree in the US. We recognize that within the sub-specialty fields of public health (e.g., environmental health, health management and policy, nutrition, health education, and others), there are long-standing undergraduate, graduate, and post-graduate degree programs. We did not include these for a variety of reasons, including that many are driven by external licensing and credentialing criteria and distinct academic accrediting bodies. Such programs unquestionably have experience and examples that contribute to the issues raised by the papers in this volume.

Second, we also recognize that public health can be taught as a secondary field of clinical disciplines, including medicine, nursing, veterinary medicine, and dentistry. This volume does not explore interrelated curricula or dual degree pedagogy. Finally, the methods by which public health is taught and practiced in the US may be quite different from how it is taught and practiced in other countries. We have left all of these expansive issues for future discussions.

## Overview

The articles comprising the eBook on UGPH are organized according to four themes.

### Theme I

Theme I presents articles that describe the recent history and background of undergraduate education for public health.

Riegelman, Albertine, and Wykoff, each of them a leader with direct experience in promoting UGPH, describe the initiatives that have led to the recent evolution of undergraduate education for public health in the US (3).

With a nationwide perspective on emerging public health education, Albertine views the collaboration between academics and clinicians from across disciplines as positive (4). However, she questions if programs are not “mini MPH” degrees, then what are they, and what prior knowledge is available to guide the design of these new programs? She implores educators to be “intentional” and “thoughtful” in building curricula and to take advantage of the pedagogical tools available.

Evashwick, Tao, and Arnold present results of the search of literature on UGPH published in peer reviewed journals between 2004 and 2014, with a total of 24 articles appearing (5). They conclude with a call for increased sharing of information through professional literature, as well as the need for research and evaluation studies on UGPH.

### Theme II

Theme II contains descriptions of existing programs, their exemplary features, and “lessons learned.”

Stoots and colleagues describe the undergraduate curriculum in public health that the University of East Tennessee has developed. As one of the oldest undergraduate public health programs in the US, it places particular emphasis on aligning the curriculum with the needs of the local workforce (6). White describes the undergraduate program developed at Tulane University, receiving a major push from another external force – Hurricane Katrina and its impact on the university and its students (7). The burgeoning new undergraduate program stimulated changes to the long-established graduate programs.

Griffin, DiFulvio, and Gerber describe the use of undergraduate public health peer advisers for a fast-growing undergraduate public health program at the University of Massachusetts Amherst (8). Peer advisers play a valuable role in supporting the program advisers in one of the University’s fastest growing programs, a B.S. in Public Health Sciences. Peer advisers do not replace the role of a professional academic adviser, but they can support the professionals and the students in the program. Being a peer adviser teaches leadership, communication skills, strategies for improving student success, and fosters personal and professional development.

Yeatts explains the importance of utilizing active learning strategies in an undergraduate introductory public health course (9). Covering public health disciplines such as epidemiology, biostatistics, health behavior, nutrition, maternal and child health, environment, and health policy, students develop an appreciation of these fields through practical exercises that give real world public health experiences both in and outside the classroom.

Nelson-Hurwitz and Tagorda describe a three-course capstone series that is a key component of the Bachelor of Arts in Public Health at the University of Hawaii at Manoa (10). Spread over three semesters, the course engages students in development and execution of a project to apply academic skills to a real-world problem.

Riegelman and Wilson consider the contribution of community colleges in training future public health professionals (11). They report on a two-phase study. Phase one consisted of an Expert Panel that developed a series of Foundation and Consensus Statements that reflect what public health and community college educational organizations could do together. Phase two is the development of “prototype curricular models for Public Health: the Generalist, the Specialist, and the Health Navigator model” for community colleges.

### Theme III

Theme III focuses on curriculum issues, including the relevancy of accreditation.

The seven articles address broader curriculum design, including accreditation, employment opportunities, and articulation with graduate programs. The authors reflect varied perspectives on whether undergraduate public health degrees should focus on preparing students for further study or direct entry into the public health workforce. Regardless, the authors all agree that any program needs to carefully assess its student market and the goals for the program, whether preparation for the job market or for further professional or academic study.

Friedman and Lee provide a framework for a baccalaureate curriculum in public health, including general education requirements as defined by the institution, core public health classes taken by all public health students, domain-specific classes if students can choose specific concentrations or courses offering more depth for a generalist degree, electives, and field experience (12). In the context of a professional orientation to the program, Friedman and Lee also advocate for professional skills and norms such as leadership, respect for others, cultural competency, team work, and conflict resolution.

Lee and Friedman acknowledge that undergraduate public health programs are relatively unusual in that they have evolved after graduate programs in the same discipline, noting that this history has introduced challenges in the articulation between programs (13, 14). They discuss the challenges of related content taught at graduate and undergraduate levels with varying depth, breath, and competencies, especially for faculty teaching at both levels and students who progress from undergraduate to graduate programs. Options to address potential duplication vary by institutional policy but might include dual degree agreements (e.g., 4 + 1 or 3 + 2 programs), course waivers based on content analysis, early matriculation, enrollment in graduate courses while still an undergraduate, tailored program requirements to minimize course duplication, waiver of specific requirements, and direct matriculation to doctoral programs.

Wykoff and colleagues present results from alumni and employer surveys at East Tennessee State University to support their view that baccalaureate degrees in public health should be developed as professional degrees preparing students for the workforce (15). About one-third of the BSPH graduates found employment in a healthcare delivery organization, with nearly half reporting other employment; graduates report general satisfaction with their preparation for the workplace.

Holsinger and colleagues reviewed 19 programs from 17 accredited schools of public health offering undergraduate degrees (16). Across the 13 BA and BS programs, public health content comprised 29–30% of the typical 120 semester hours of coursework. In contrast, for the six BSPH or BPH programs, public health content represented an average of 52 semester hours or 43% of the curriculum; the authors note that these numbers represent minimum required courses without consideration of potential electives in public health. Holsinger compares these numbers to the typical MPH curriculum, noting the interpretation must be tempered by the different levels of education, and acknowledging that the baccalaureate graduates may be competing for entry level public health jobs previously filled by MPH graduates.

From the perspective of lengthy experience with accreditation of graduate public health education, King and Petersen describe the development of accreditation criteria for baccalaureate programs not affiliated with accredited schools or programs of public health as a coordinated approach to assure quality in undergraduate public health education without restricting to professional or academic programs (17). Informing this development were multiple conversations involving public health and educational leaders to develop consensus about content and structure. Commonalities beyond public health core content include personal and social responsibility, determinants of health and disease, and experiential opportunities.

Tarasenko and Lee examine three major college directories and the Association of Schools and Programs of Public Health website to identify undergraduate degrees in public health, and then compare the specifics of the curriculum with the universities’ catalogs (18). In contrast to much larger numbers reported by King and Petersen (17) and Holsinger and colleagues (16), Tarasenko and Lee identify only 54 programs that offer a general degree in public health. They contrast this with the apparent popularity of public health among universities and undergraduate students, concluding that undergraduate education for public health is still in its infancy, both in terms of curriculum development and broad marketing efforts and general awareness.

### Theme IV

Theme IV offers insights into the relationship between formal academic training and careers in the public health or healthcare workforce.

Tilson, Bender, and Kronstadt describe the new accreditation for state and local public health departments, their responsibilities for “maintaining a competent workforce,” and how UGPH can work with government public health departments to cultivate the workforce for the future (19). Learning from existing collaborations among and between the academic enterprises and state, local, tribal, and territorial health departments should prove useful as accreditation is more uniformly achieved across the US.

Martin discusses how education in public health can be applied to careers in healthcare delivery settings (20). The author presents a compelling case that as health care increasingly adapts to a value-based and population-oriented delivery model, a new generation of professionals will require skills and abilities to communicate and collaborate in social systems that grow increasingly complex over time. UGPH provides a foundation for management and clinical careers, as well as those emphasizing public health.

Lee reviews the book *Career Planning in Public Health*, which identifies and examines 101 employment options within the sphere of public health (21). The author describes the strengths and weaknesses of the book, and provides useful alternate sources of information intended to complement the book review itself. Readers with an interest in navigating potential career pathways will find the article a useful orientation to the diverse and often puzzling portals of entry into a rewarding and aspirational public health career. This could well be used by university faculty, counselors, or students themselves to explore the possible careers that build upon a foundation of formal education in public health.

## Conclusion

We would like to thank all of those who contributed to this Research Topic as authors, editors, review editors, and thoughtful colleagues. Practitioners, as well as academicians, participated in shaping the articles that are showcased here. Many others are working in the field as teachers, practitioners, public health leaders, and students. The need for additional data is evident; rigorous evaluation and research studies are needed. The ideas articulated here hopefully contribute to identifying the issues that warrant analysis. We are all striving to create a public health system and public health workforce that will enable the US to maximize the health status of its communities. We hope the information and ideas shared here will further this goal.

## Conflict of Interest Statement

The authors declare that the research was conducted in the absence of any commercial or financial relationships that could be construed as a potential conflict of interest.
